# Emerging concepts in drug discovery for cancer therapy

**DOI:** 10.1002/1878-0261.13325

**Published:** 2022-11-02

**Authors:** Matilde Murga, Oscar Fernandez‐Capetillo

**Affiliations:** ^1^ Genomic Instability Group Spanish National Cancer Research Centre (CNIO) Madrid Spain; ^2^ Science for Life Laboratory, Division of Genome Biology, Department of Medical Biochemistry and Biophysics Karolinska Institute Stockholm Sweden

These are exciting times to be involved in biomedical research. The transition from a scientific hypothesis to a testable therapy is now faster than ever, and new technologies that facilitate drug development are constantly emerging. The rapid development of COVID‐19 vaccines provides a clear example of the current pace of drug discovery and its clinical implementation. Importantly, the wide availability of technologies that speed up drug development has also reached oncology, and investigators can now consider several independent strategies when looking to develop a new therapy. This thematic issue provides an overview of recent advances in cancer drug discovery, in areas such as fragment‐based drug development, targeting the DNA damage response or the MYC oncogene, senolytic therapies and computational tools that facilitate drug discovery and the selection of treatments in personalized medicine. We are confident that reading these reviews will help those interested in the development of cancer therapies get a broader and updated view of available opportunities and challenges.

Research performed primarily over the last century has dramatically improved cancer prognosis. As an example, cancer survival in the UK has doubled in the last 40 years and, today, half of the cancer patients survive for 10 or more years from diagnosis [1]. Due to the impressive success of immunotherapy based on immune checkpoint blockade [2] or CAR‐T cells [3] in tumors of poor prognosis, there is a growing interest in biological therapies. Accordingly, the percentage of biologics license applications (BLAs) granted approval by the Food and Drug Administration (FDA) has increased during the last decade, reaching 28% in 2021 [4]. Despite this recent surge of BLAs, the toolbox for developing chemical therapies is also expanding.

One of the areas of drug discovery that is currently going through a ‘renaissance’ period is that of fragment‐based drug discovery (FBDD). In contrast to large and unbiased high‐throughput screens (HTS), which have dominated the drug discovery landscape over the recent decades, FBDDs use smaller libraries to discover low‐molecular weight (≤ 300 Da) molecules with high affinity for a given target, which can then be grown into efficient drugs through medicinal chemistry. Importantly, FBDD has notorious examples of success such as the development of venetoclax as the first drug targeting a protein–protein interaction [5], or the recent approval of sotorasib as the first inhibitor targeting a mutant version of the KRAS oncogene (KRAS G12C) [6]. While RAS oncoproteins were often considered ‘undruggable’, the key development that enabled the development of sotorasib was the discovery of an actionable pocket on the KRAS‐G12C variant by the group of Kevan Shokat in 2013 [7]. These exciting examples have revitalized the interest on FBDD. In this context, the review by Marta Bon and colleagues [8] defines key aspects that must be considered when approaching FBDD. Particular detail is placed on the importance of defining a chemical library with sufficient diversity and properties that facilitate the subsequent development of the initial hits. As in many other fields, this is now substantially facilitated by computational methods, which are also summarized. The review also covers other important advances in the field such as in the technologies available for hit‐to‐lead development, or on the various approaches for identifying covalent binders.

The next three reviews of this thematic issue focus on cancer therapies that target the DNA damage response (DDR). Genotoxic chemotherapies were the first chemical cancer therapies to be developed, and are still one of the most widely used strategies for the treatment of patients in oncology. An evolution to this approach was the development of inhibitors targeting enzymes that participate in the repair or signaling of DNA damage. Such approaches could be particularly efficacious in tumors with defects in DNA repair. Arguably, the most exciting discovery in this regard came in 2005 with the independent observation by two groups that poly(ADP‐ribose) polymerase (PARP) inhibitors were preferentially toxic to cancer cells carrying *BRCA1* or *BRCA2* mutations [9, 10]. Today, PARP inhibitors are a clinical reality and their use has been extended to tumors with deficiencies in homologous recombination (HR) [11]. This story of success triggered an intense effort oriented to optimize the use of PARP inhibitors, as well as to discover new synthetic lethal interactions that could be exploited for cancer treatment. Other examples now include ATR kinase inhibitors for the treatment of tumors with high levels of replication stress (RS) [12], already being explored in clinical trials, or the essentiality of the WRN helicase in tumors with microsatellite instability (MSI) [13], which has triggered a race for the discovery of WRN inhibitors.

Along this theme, Jordan Wilson and Joanna Loizou review all available approaches that exploit variations of CRISPR‐based genetic screens [14], and how these can be used for the discovery of new genetic interactions that can be pharmacologically targeted. In addition, they summarize recent developments on the discovery of chemical DNA repair inhibitors including proteolysis‐targeting chimeras (PROTACs) [15]. Next, Rudd and Helleday review their efforts in targeting enzymes involved in dNTP metabolism, particularly on the enzymes MTH1, MTHFD2 and SAMHD1 [16]. The efficacy of this approach is exemplified by the long use of anti‐folates, the oldest efficacious cancer chemotherapy, discovered by Sidney Farber and colleagues back in 1948 [17]. Today, many clinically approved therapies target nucleotide metabolism in various tumors, yet toxicities, resistance mechanisms and drug interactions ultimately limit their efficacy. Rudd and Helleday propose that such barriers may be overcome via multiple independent studies for a better determination of the mechanism of action of metabolic enzyme inhibitors. Finally, Baxter et al. [18] review the mechanisms of resistance to DNA repair inhibitors, a very active area of research which should help to define better drug combinations and focus the use of DNA repair inhibitors onto patients that are more likely to respond. The manuscript mainly focused on resistance to inhibitors of PARP, ATR, PolΘ or WRN, summarizing the large number of mutations that have been shown to modify the synthetic lethal interactions involving these enzymes, as well as delineating the strategies as to how these resistances could be overcome. An important point raised by the authors is that, since many of these interactions have been found through genetic screens conducted *in vitro*, the relevance in the clinic for most of them remains to be seen.

Indirectly related to therapies targeting cells harboring DNA damage, are treatments aiming to eliminate senescence cells, also known as senolytics, here reviewed by Laura Bousset and Jesus Gil [19]. Senolytic drugs have raised significant interest in the field of cancer therapy, mainly for their potential to clear senescent cells that accumulate upon a previous treatment with a pro‐senescent drug (known as the ‘one‐two‐punch’ approach [20]). In addition, selective elimination of senescent cells in mouse models was associated with increased longevity and widespread beneficial effects on age‐related pathologies, raising further excitement about the potential applications of senolytic drugs [21, 22, 23]. Despite the growing interest in this field, it is relatively new and many aspects need to be further addressed: what is the contribution of non‐tumoral senescent cells to cancer progression? Can the elimination of senescent cells favor tumor development in some instances? Can we develop senomorphic drugs to modulate the senescence‐associated secretory program (SASP)? Moreover, and while SA‐β‐galactosidase activity is a useful hallmark of senescence in research studies, a clinical biomarker for senescent cells is still needed. Still, definitely an area to watch for in the coming years.

Next, Giulio Donati and Bruno Amati review efforts and challenges of targeting *c‐*MYC (hereafter, MYC) for cancer therapy [24]. The MYC transcription factor governs most growth and survival pathways in cells, and is overexpressed in multiple cancers. In addition, recent studies have revealed that increased MYC activity correlates with drug resistance. Accordingly, targeting MYC has been an important aim for the cancer research community for decades. However, given that MYC is a transcription factor lacking catalytic activity, targeting MYC remains a challenge. Their article describes the efforts that have been dedicated to generating MYC inhibitors such as chemicals targeting the MYC–MAX interaction, drugs that reduce MYC‐dependent transcription such as BRD inhibitors, or MYC‐targeting peptides such as OMOMYC. In addition, the authors discuss the approach of targeting specific vulnerabilities that emerge upon MYC overexpression, such as an increased dependence on the DDR or on mitochondrial activity. Importantly, the work also raises a word of caution on the potential selectivity of currently available drugs targeting MYC, and highlights the need for better and more specific drugs.

Finally, one of the main scientific revolutions that is inevitably transforming cancer research is the advance of computational approaches. Today, the development and selection of treatments is greatly facilitated by a constellation of bioinformatic tools, which are being developed at an ever‐increasing speed. Ourselves, we are increasingly becoming attracted to integrating computational methods for most of our own projects related to drug discovery, and thus wanted to end this thematic issue with a dedicated chapter. María Jimenez‐Santos et al. provide a very comprehensive overview of bioinformatic tools that can be used to infer the mechanism of action of a drug, stratify patients for personalized cancer medicine, or follow the evolutionary trajectories of cancer cells in a given tumor. The review also highlights the need for standardized methods that can facilitate the integration of these tools in clinical practice. Nevertheless, given the impressive developments in this area, we are confident that the future of cancer research will be progressively incorporating computational support.

Coordinating this thematic issue has been a pleasure. Part of the research in our laboratory has been dedicated to drug discovery, although we have favored phenotypic HTS for our projects, such as for the development of ATR inhibitors [12, 25]. Having read these reviews has given us a much broader view on opportunities in other areas, which will likely influence our own choices in the future. We want to end by wholeheartedly thanking the authors of these reviews for their work, and hope the readers find these reviews as useful as we did.

## Conflict of interest

The authors declare no conflict of interest.

## Author contributions

MM and OF wrote the MS.
